# Comparison of Landmark- and Outline-Based Geometric Morphometrics for Discriminating Mosquito Vectors in Ratchaburi Province, Thailand

**DOI:** 10.1155/2018/6170502

**Published:** 2018-11-06

**Authors:** Tanawat Chaiphongpachara

**Affiliations:** College of Allied Health Science, Suan Sunandha Rajabhat University, Samut Songkhram 75000, Thailand

## Abstract

It is often challenging to identify mosquito vectors in the field based on morphological features due to their similar morphologies and difficulties in obtaining undamaged samples but is required for their successful control. Geometric morphometrics (GM) overcomes this issue by analyzing a suite of traits simultaneously and has the added advantages of being easy to use, low cost, and quick. Therefore, this research compared the efficiency and precision of landmark- and outline-based GM techniques for separating species of mosquitoes in Huay Nam Nak village, Ratchaburi Province, Thailand. This research collected 273 individuals belonging to seven species:* Anopheles barbirostris*,* An. subpictus*,* Culex quinquefasciatus*,* Cx. vishnui*,* Cx. whitmorei*,* Aedes aegypti*, and* Ae. albopictus*. Both landmark-based and outline-based GM techniques could identify malaria vectors in this area to the genus level successfully and were also very effective for identifying the malaria vectors* Anopheles* spp. and the dengue vectors* Aedes* spp. to the species level. However, they were less effective for distinguishing between species of* Culex*. Therefore, GM represents a valuable tool for the identification of mosquito vectors in the field, which will facilitate their successful control.

## 1. Introduction

Mosquitoes (Diptera: Culicidae) are medically important small insects that transmit many diseases to humans, particularly in tropical and subtropical regions [1]. Globally, there are 3,400 species of mosquitoes in 42 genera, many of which are important vectors of pathogens [2]. Mosquito-borne diseases, which include dengue fever (DF), chikungunya, malaria, Japanese encephalitis, and filariasis [3], account for 17% of all infectious diseases worldwide and are responsible for approximately 1.4 million deaths per year [4], making them a serious public health issue.

Mosquito-borne diseases are one of the most important health concerns in Thailand. According to the Bureau of Epidemiology of Thailand, DF had the highest incidence rate in 2014 at 35.67 cases per 100,000 people, followed by malaria (17.48), chikungunya (0.29), filariasis (0.03), and Japanese encephalitis (0.02) [5]. Ratchaburi Province on the western border of Thailand has one of the highest incidences of mosquito-borne diseases, particularly DF and malaria, with morbidity rates of 30.68 and 8.95 cases per 100,000 population, respectively. Therefore, it shows that Ratchaburi Province has an epidemic of mosquito-borne diseases still, making it important that control methods are found to reduce the number of patients in this area.

Mosquito control is one important strategy for controlling mosquito-borne disease epidemics [6–8]. However, since different species of mosquitoes have different characteristics, such as behaviors, breeding sites, and epidemiologies, a knowledge of which vector species are present in the area [9, 10] and the epidemiological patterns of disease transmission is required to ensure the planned control method is appropriate [11, 12]. The It is can be challenging to identify mosquito vectors in the field based on morphological features because many species are cryptic species, sibling species, or isomorphic species with similar morphologies [13]. Furthermore, it is more difficult to obtain a mosquito sample in the field than in the laboratory because the external characteristics are often damaged during trapping and transportation [14]. One solution to this is the use of high-efficiency molecular techniques for mosquito identification. However, these have very high associated costs [14, 15], making them unsuitable for use in the field where many samples are collected. Thus, a new technique for identifying mosquito vectors in the field is required.

Geometric morphometrics (GM) is a fast, inexpensive technique [14, 16] used to analyze the size and shape of individuals based on a suite of traits. GM has been applied widely in a number of fields, including entomology (e.g., mosquito [17], blow fly [18], bee [19], and eggs of Triatominae [20]). Furthermore, several studies have used the GM method to classify species and to examine variation among medically important mosquitoes that are morphologically similar or sibling species [14, 15, 21–24]. GM analyses can be conducted using landmark- or outline-based methods [13], each of which has different advantages depending on the characteristics and specificity of the sample. The landmark-based approach uses the coordinates of landmarks to analyze the morphology [23] and is popular in the field of medical entomology, with many studies having shown it can distinguish different species of mosquito vectors successfully, such as* Anopheles* spp. and* Aedes *spp. [14, 21, 23]. By contrast, the outline-based approach uses contour data [25] and has also been shown to be useful for identifying some mosquito species, such as* Aedes scutellaris* [14]. However, surprisingly, the specificity with which mosquito vectors can be identified differs between the two techniques [14, 23] and, therefore, a comparison of their performance in the field is required before they can be used.

Thus, the aim of this study was to compare the efficiency of landmark- and outline-based GM techniques to separate species of mosquito vectors in Huay Nam Nak village in Ratchaburi Province, Thailand, an endemic area for mosquito-borne diseases. The result from this research will serve as a guideline for choosing the best GM technique for the identification of vectors in the field in Thailand to facilitate the control of vector-borne diseases.

## 2. Materials and Methods

### 2.1. Study Site and Mosquito Collection

**Figure 1:**
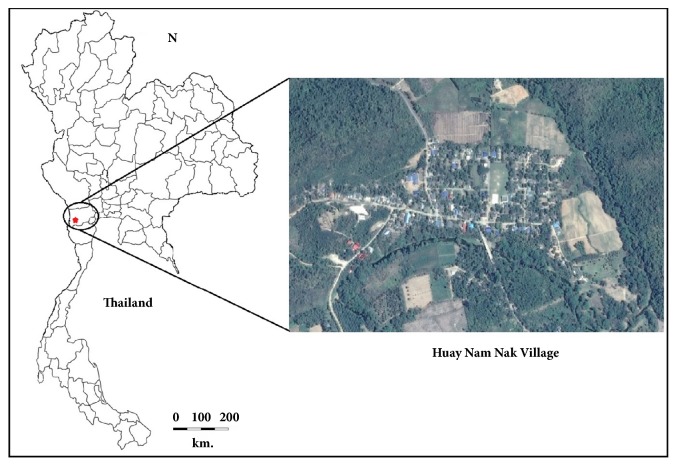
Location of the study site.

Mosquitoes were collected from Huay Nam Nak village (13°22′36.0′′N, 99°16′34.9′′E) in the Suan Phueng District of Ratchaburi Province, Thailand, during June to August 2015 (Figure 1). The study site consisted mainly of wooden houses and agricultural fields, with streams flowing through and mountains and hilly forests surrounding the village.

Three Mosquito Magnet® Independence Mosquito Traps (Woodstream Corporation, USA) were placed approximately 5 meters away from houses in the village for 24 hours (6:00 am to 6:00 am of the following day) per week over the 3-month study period to collect nocturnal and diurnal mosquito vectors. Mosquito samples were collected from each trap in the morning (6.00 am) and transported to the laboratory of the College of Allied Health Science, Suan Sunandha Rajabhat University, Samut Songkhram Province for morphological identification. Female mosquitoes were then morphologically identified to species using the illustrated keys to the mosquitoes of Thailand [26].

### 2.2. Mosquito Wing Preparation

Only the right wings of female mosquitoes were analyzed by GM. The right wing of each individual was dissected under a Nikon AZ 100M stereomicroscope (Nikon Corp., Tokyo, Japan) and placed between a glass microscope slide and coverslip using Hoyer's solution as a mounting medium. Then, all of the wing samples for each species were photographed using a Nikon DS-Ri1 SIGHT digital camera connected to a Nikon Eclipse E600 microscope (Nikon Corp., Tokyo, Japan) under 40× magnification alongside a 1-mm scale bar and were analyzed using the Collecting Landmarks for Identification and Characterization (CLIC) Program. Since the aim of this study was to compare the effectiveness of landmark- and outline-based GM for species discrimination, the same set of wing pictures was used for each species in the two analyses outlined below.

### 2.3. Landmark-Based GM Analysis

**Figure 2:**
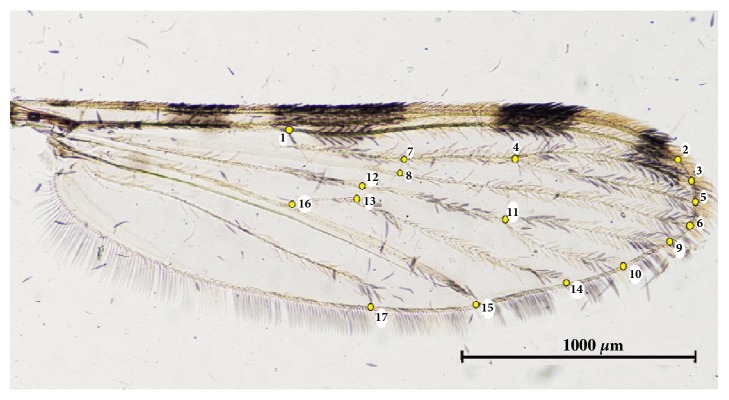
Position of the 17 landmarks on the mosquito wing used for the landmark-based GM analysis.

A total of 17 landmarks were selected based on the ease with which they could be plotted across all mosquito species and their low likelihood of being damaged, e.g., by wing vein intersection. The positions of these landmarks on the wing of each individual were digitized (Figure 2). The wing size was then computed as the centroid size (CS), which is the square root of the sum of the squared distances between each individual landmark and the center of the landmark configuration [14, 27]. The wing shape variables (partial warps [PW]) were computed as principal components (PCs) of the PW (known as relative warps [RW]) after Generalised Procrustes Analysis (GPA) (the statistical procedure of superimposition) and discriminant analysis (DA) (or Canonical Variate Analysis [CVA]) was then used to analyze the shape variables for the separation of each species.

### 2.4. Outline-Based GM Analysis

**Figure 3:**
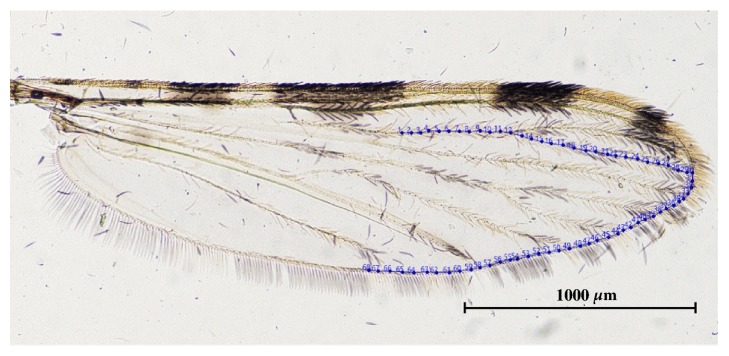
Digitized contour of the lower section of a mosquito wing used for the outline-based GM analysis.

The outline used for analysis was constructed using coordinates along the contour of the lower part of the mosquito wing (Figure 3). This analytical contour is the most complete part of the wing in all mosquitoes and also lacks thick scales and is difficult to tear. The wing size was estimated as the length of the perimeter of the contour, which was separate from the set of shape variables used. The shape variables were constructed using the normalized elliptic Fourier coefficients (NEF).

### 2.5. Statistical Analysis

Prior to conducting the GM analyses, this research assessed the quality and measurement error of the digitized landmarks and digitized contour used for the landmark-based and outline-based GM analyses, respectively, by calculating the repeatability index (R). The repeatability of each coordinate was tested for 70 randomly selected wings (10 wings per species). In order to do this, measurements of the coordinates used in the original wing photographs were repeated to give a total of 140 images. The repeatability was then computed based on an ANOVA design. Prior to analysis, the error was reduced by averaging the two digitizations [23, 25].

The variation in wing CS (in mm) of mosquito samples each species was illustrated by quantile plots between P 25% and P 75%. Differences in the wing CS (for landmark-based GM) and the length of the perimeter (for outline-based GM) among species were analyzed using nonparametric permutation tests (1,000 runs) with Bonferroni correction test and a significance level of* p* < 0.05.

Wing shape variation among species was also visualized by superimposition the mean landmark configurations using Procrustes superimposition in the landmark-based analysis and using EFA in the outline-based analysis. The shape variables for each species were separated using DA and displayed as a factor map. The Mahalanobis distance was calculated from DA to assess the degree of similarity between population and differences in the Mahalanobis distance (i.e., wing shape) among species were computed using nonparametric permutation tests (1,000 runs) with Bonferroni correction test and a significance level of* p* < 0.05. Then, validated reclassification, whereby each individual was allocated to its closest group according to the Mahalanobis distance without being used to help determine the group center, was performed [23], and a single linkage hierarchical classification tree was created based on the bootstrap technique on input data within species, according to Morales Vargas et.al. [28] and visualized using XYOM.

Finally, since size and shape are not independent attributes, this research examined the relationship between the two (the allometric effect) also using linear correlation [13] after the GM analysis of the size variables.

### 2.6. Software

Both the landmark- and outline-based GM analyses were performed using the CLIC package version 97, which was developed by Professor Jean-Pierre Dujardin [13, 29] and is freely available at http://xyom-clic.eu/. Various modules of the CLIC package were used, including the COO module to digitize the landmarks or pseudolandmarks. The TET module was used to transform the data for analysis. The MOG and FOG modules were used to construct the size and shape variables, perform principle component analyses (PCA) and DA, compute Procrustes distances, and create quantile plots in the landmark- and outline-based analyses, respectively. The VAR module was used to analyze allometry and the statistical significance of differences in the size variables among species. The PAD module was used to analyze the statistical significance of differences in the shape variables among species. The single linkage hierarchical classification tree as built by the recent online morphometric package, XYOM (https://xyom.io), was compared to the UPGMA tree as computed by the R software (https://cran.r-project.org/).

## 3. Results

This research collected 273 mosquitoes belonging to seven species within three genera (Table 1).

### 3.1. Repeatability

The two sets of repeated measurements from the same images used in the landmark- and outline-based GM analyses showed good repeatability scores for both size and shape. In the landmark-based analysis, the repeatability of the CS was 0.98, while in the outline-based analysis, the repeatability of the perimeter length was 0.97.

### 3.2. Allometry

This research found a weak relationship between size and shape, with the allometric residuals of the discriminant factors explaining 8% of the variation in the landmark-based GM and 5% of the variation in the outline-based GM. However, since both of the components are important for species identification, neither was removed from the GM analyses.

### 3.3. Wing Size

**Figure 4:**
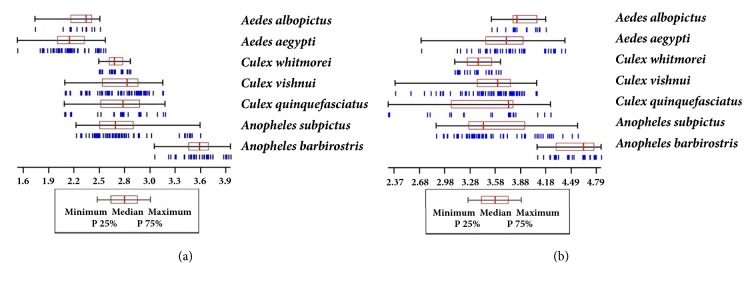
Variation in (a) the wing centroid size used in the landmark-based analysis and (b) the perimeter length of the contour used in the outline-based approach in mosquito species. The plots in each panel show the 25th and 75th quartiles and the median.

In the landmark-based analysis,* An. barbirostris *had the largest wings (mean = 3.54 mm) and* Ae. aegypti *had the smallest (mean = 2.19 mm) (Table 2 and Figure 4). In addition,* An. barbirostris* had significant intraspecific variation in mean CS that was the highest among all species.

In the outline-based analysis,* An. barbirostris *again had the largest wings (mean = 4.51 mm) while* Cx. whitmorei* had the smallest (mean = 3.39 mm). Both* An. barbirostris *and* Ae. albopictus *exhibited significant intraspecific variation in the mean perimeter length of the contour.

### 3.4. Wing Shape

**Figure 5:**
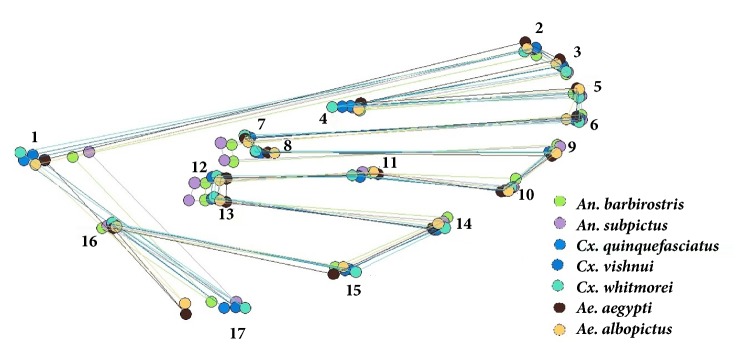
Superimposition of the mean landmark configurations of the wings for the seven mosquito species.

**Figure 6:**
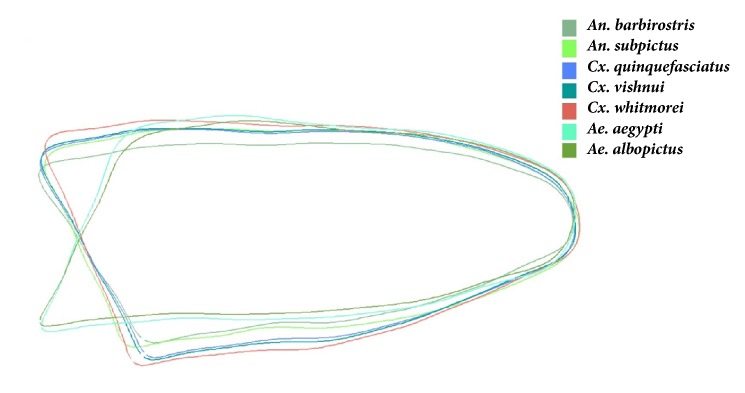
Superimposition of the outlines of the wings for the seven mosquito species.

Superimposition of the mean wing shapes of each mosquito species using Procrustes superimposition showed the positions of landmarks 1, 7, 8, 12, 13, and 17 varied among species in the landmark-based analysis (Figure 5), and the outlines of* Ae. aegypti *and* Ae. albopictus* were distinct from the other species in the outline-based analysis (Figure 6).

**Figure 7:**
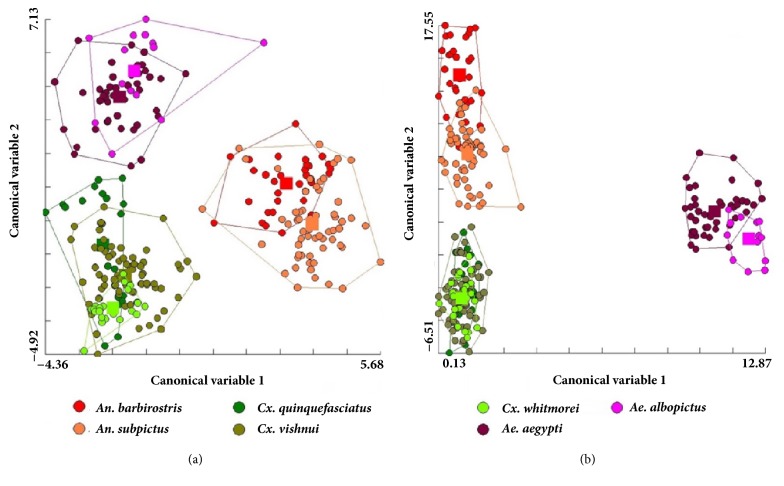
Factor maps from (a) landmark- and (b) outline-based discriminant analysis.

Comparison of the factor maps derived from DA showed both the landmark- and outline-based GM analyses gave very similar results, particularly at the genus level (Figure 7). Based on the Mahalanobis distances, wing shape was significantly different among all species in both the landmark- and outline-based GM analyses (nonparametric permutation test, 1,000 cycles,* p *< 0.05; Table 3).The greatest Mahalanobis distances were between* Ae. aegypti* and* An. subpictus* (8.86) in the landmark-based analysis and between* Ae. albopictus* and* An. barbirostris* (22.67) in the outline-based analysis.

**Figure 8:**
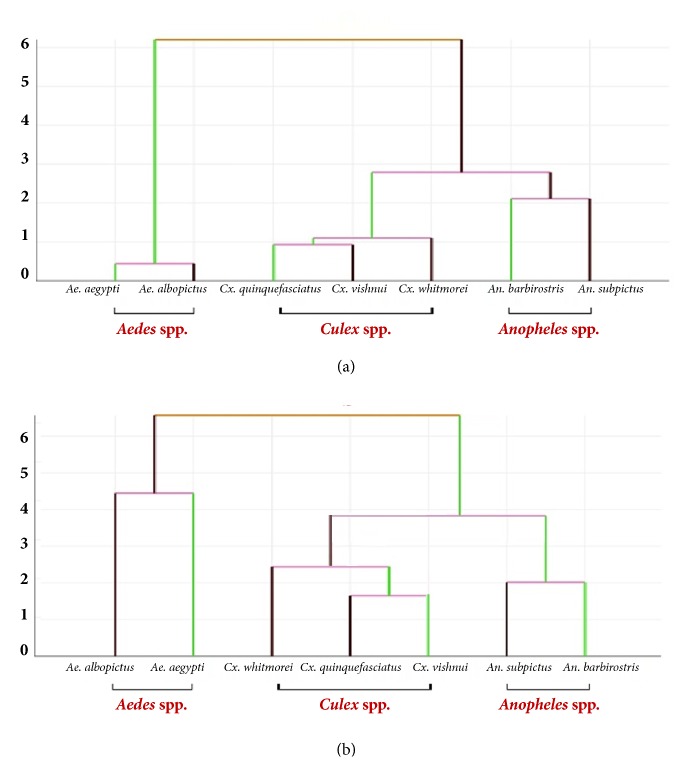
Single linkage hierarchical classification tree for the (a) landmark and (b) outline-based geometric morphometric analyses.

The validated reclassification scores were high (>80% accuracy) for five species using the landmark-based approach (*An. barbirostris *[96%],* An. subpictus* [81%],* Cx. whitmorei *[91%],* Ae. aegypti *[88%], and* Ae. albopictus *[90%]) and four species using the outline-based approach (*An. barbirostris *[93%],* An. subpictus *[86%],* Cx. vishnui* [85%],* Ae. aegypti *[86%], and* Ae. albopictus *[95%]). The validated reclassification scores were lowest for* Cx. vishnui* with the landmark-based approach (65%) and for* Cx. quinquefasciatus* with the outline-based approach (42%) (Table 4). Single Linkage Hierarchical classification trees separated each species of mosquito (Figure 8). The trees that were produced using the two different approaches were very similar.

## 4. Discussion

This research analyzed 273 wings across seven species of mosquitoes using two different GM approaches. Mosquito wings are almost bidimensional and relatively rigid, which reduces mistakes when digitizing them for GM analysis [16], as reflected by the good repeatability scores for both size and shape in the present study.

### 4.1. Wing Size

This result found few differences between the landmark- and outline-based analyses in terms of the wing CS, with both methods showing* An. barbirostris* and* Aedes* spp. had significantly larger and smaller wings, respectively, than the other species. However, these species also exhibited significant intraspecific differences in size, likely due to variation in factors such as temperature, humidity, and food availability [15]. Because of such variation, it has been suggested previously shape is more appropriate than size for distinguishing among morphologically similar species, and is also more informative in terms of the genetics and evolution of organisms [15, 16, 23]. However, wing size is also useful in the initial identification of species, particularly since some species of mosquitoes are larger than many other species; for example,* An. barbirostris* was clearly larger than* Culex* species in this study. Similarly, Stanford et al. [29] found wing beat frequencies, which mediate assortative mating, are related to size and consequently are unique for each species.

### 4.2. Wing Shape

The shape and venation of mosquito wings are unique characteristics that can be used to separate different genera and species [14]. This research found little difference between the landmark- and outline-based methods when considering the wing shape of mosquitoes. For both methods, factor maps derived from DA showed there was no overlap between genera but some overlap between species (Figure 7). However, wing shape was found to be significantly different among all seven species using both methods. Similarly, Wilke et al. [30] found GM was a good tool (100% accuracy) for species identification at the genus level for* Aedes*,* Anopheles*, and* Culex*, but was also efficient at the subgenus and species levels. In both GM analyses, the reclassification scores were higher for* Anopheles *spp. and* Aedes *spp. (>80% accuracy in all species) than for* Culex *spp., with the exception of* Cx. whitmorei* for the landmark-based analysis (91%) and* Cx. vishnui* for the outline-based analysis (85%). Consistent with this, the single linkage hierarchical classification trees clearly separated the three genera in the landmark- and outline-based analysis (Figure 8(a)). Superimposition of the mean landmark configurations of each mosquito species showed landmarks 2, 3, 5, 6, 7, 9, and 10 were in similar places for* Culex *spp. (particularly* Cx. quinquefasciatus *and* Cx. vishnui*) and the other species (Figure 5).

These findings show GM can be used successfully to classify* Anopheles *spp., which are often implicated in malaria transmission*; An. barbirostris* is a suspected vector of* Plasmodium *spp. in Thailand [31], while* An. subpictus *is not considered a malaria vector in Thailand but is a vector in Sri Lanka [32]. GM is also useful for identifying* Aedes *spp., including* Ae. aegypti *and* Ae. albopictus*, which are dengue and chikungunya vectors, respectively [14]. However, GM had low classification rates for* Culex *spp., including* Cx. quinquefasciatus*,* Cx. vishnui*, and* Cx. whitmorei*, which have been incriminated as Japanese encephalitis and filariasis vectors [26].

Our findings demonstrate both GM approaches can be used to identify mosquitoes in the study area, particularly to the genus level. Currently, the landmark-based GM approach is the most popular for helping to identify species and investigating variation among vectors [14, 21, 23] as it is less time-consuming, requiring the definition of only a few analytical points for analysis, and is a powerful approach. By contrast, few studies use the outline-based GM approach because it requires a lot of time and samples than the landmark-based approach. However, this approach has the advantage of not requiring the use of specific locations [25]. Furthermore, this research found that the outline-based GM approach was better and more effective at discriminating some species, such as* Cx. vishnui*.

## 5. Conclusions

Both the landmark- and outline-based GM methods are practical and effective for discriminating between species of mosquitoes in the study area. However, since each species of mosquito has a unique wing identity, the best method needs to be selected to suit the species occurring in a particular vector control area. GM has the advantages of being easy to use, low cost, and quick, as well as not requiring advanced entomological skills, and these advantages make it particularly attractive for use in the field to facilitate the control of mosquito vectors.

## Figures and Tables

**Table 1:** Species and numbers of mosquitoes used in the analyses.

Species	Number of mosquitoes
*Anopheles barbirostris*	31
*Anopheles subpictus*	61
*Culex quinquefasciatus*	21
*Culex vishnui*	70
*Culex whitmorei *	24
*Aedes aegypti*	45
*Aedes albopictus*	21

Total	273

**Table 2:** Mean wing centroid size and mean perimeter length of the contour in the seven mosquito species.

Species		Landmark-based approach		Outline-based approach	
	n	Mean (Min - Max) (mm)	S.D.	Mean (Min - Max) (mm)	S.D.
*An. barbirostris*	31	3.54 (3.08 - 3.88)^a^	0.19	4.51(4.06 - 4.79)^a^	0.21
*An. subpictus*	61	2.74(2.25 - 3.56)^b^	0.34	3.58(2.92 - 4.52)^b,c^	0.42
*Cx. quinquefasciatus*	21	2.71(2.13 - 3.19)^b^	0.33	3.46(2.37 - 4.22)^b^	0.53
*Cx. vishnui *	70	2.70(2.13 - 3.17)^b^	0.26	3.51(2.45 - 4.05)^b^	0.34
*Cx. whitmorei*	24	2.66(2.49 - 2.82)^b^	0.11	3.39(3.13 - 3.65)^b^	0.17
*Ae. aegypti*	45	2.19(1.63 - 2.56)^c^	0.21	3.71(2.75 - 4.37)^c^	0.35
*Ae. albopictus*	21	2.30(1.82 - 2.50)^c^	0.15	3.88(3.54 - 4.16)^d^	0.19

Species with different superscripts letters had significantly different wing sizes at *p* < 0.05. Min, minimum; Max, maximum; S.D., standard deviation.

**Table 3:** Mahalanobis distances of wing shape among the seven mosquito species.

	Mahalanobis distances						
	**AB**	**AS**	**CQ**	**CW**	**CV**	**AAE**	**AAL**
**Landmark-based approach**							
*An. barbirostris*	0.00						
*An. subpictus*	4.10*∗*	0.00					
*Cx. quinquefasciatus*	7.83*∗*	8.33*∗*	0.00				
*Cx. vishnui *	7.55*∗*	7.70*∗*	2.74*∗*	0.00			
*Cx. whitmorei*	7.91*∗*	8.12*∗*	3.92*∗*	3.10*∗*	0.00		
*Ae. aegypti*	8.11*∗*	8.86*∗*	5.20*∗*	5.96*∗*	6.68*∗*	0.00	
*Ae. albopictus*	7.85*∗*	8.75*∗*	6.49*∗*	7.38*∗*	8.19*∗*	4.47*∗*	0.00

**Outline-based approach**							
*An. barbirostris*	0.00						
*An. subpictus*	7.41*∗*	0.00					
*Cx. quinquefasciatus*	9.54*∗*	6.55*∗*	0.00				
*Cx. vishnui*	9.23*∗*	6.76*∗*	3.65*∗*	0.00			
*Cx. whitmorei*	9.80*∗*	7.32*∗*	4.75*∗*	4.22*∗*	0.00		
*Ae. aegypti*	20.05*∗*	18.63*∗*	18.74*∗*	19.06*∗*	19.33*∗*	0.00	
*Ae. albopictus*	22.67*∗*	21.55*∗*	21.38*∗*	21.60*∗*	21.71*∗*	6.04*∗*	0.00

*∗* indicates statistical significance at *p*< 0.05.

**Table 4:** Validated reclassification accuracies for the seven mosquito species.

Species	Percentage of reclassification	
	Landmark-based approach	Outline-based approach
*An. barbirostris*	96% (30/31)	93% (29/31)
*An. subpictus*	81% (50/61)	86% (53/61)
*Cx. quinquefasciatus*	66% (14/21)	42% (9/21)
*Cx. vishnui *	65% (46/70)	85% (60/70)
*Cx. whitmorei *	91% (22/24)	79% (19/24)
*Ae. aegypti*	88% (40/45)	86% (39/45)
*Ae. albopictus*	90% (19/21)	95% (20/21)

## Data Availability

The data supporting the conclusions of this article are provided within the article. The datasets generated and analyzed during the current study are available from the corresponding author upon reasonable request.
